# Omega-3 polyunsaturated fatty acids and/or vitamin D in autism spectrum disorders: a systematic review

**DOI:** 10.3389/fpsyt.2023.1238973

**Published:** 2023-08-16

**Authors:** Yuwei Jiang, Wenjun Dang, Hong Nie, Xiangying Kong, Zhimei Jiang, Jin Guo

**Affiliations:** ^1^College of Rehabilitation Medicine, Jiamusi University, Jiamusi, Heilongjiang, China; ^2^Heilongjiang University of Chinese Medicine, Harbin, Heilongjiang, China

**Keywords:** autism spectrum disorders, omega-3, vitamin D, social functioning, behavioral functioning, speech function, biomarkers changes

## Abstract

This systematic review aims to offer an updated understanding of the relationship between omega-3 supplementation and/or vitamin D and autism spectrum disorders (ASD). The databases PubMed, Cochrane Library, Web of Science, EMBASE, CINAHL, Vip, CNKI, Wanfang, China Biomedical Database databases were searched using keywords, and relevant literature was hand-searched. Papers (*n* = 1,151) were systematically screened and deemed eligible since 2002. Twenty clinical controlled studies were included in the final review. The findings were analyzed for intervention effects focusing on the core symptoms of ASD, included social functioning, behavioral functioning, speech function and biomarkers changes. The review found that the effects of omega-3 supplementation on ASD were too weak to conclude that core symptoms were alleviated. Vitamin D supplementation improved core symptoms, particularly behavioral functioning, however, the results of the literatures included in this study were slightly mixed, we cannot directly conclude that vitamin D supplementation has a beneficial effect on a specific symptom of ASD, but the overall conclusion is that vitamin D supplementation has a positive effect on behavioral functioning in ASD. Omega-3 and vitamin D combination supplementation has a good combined effect on social and behavioral outcomes in patients with ASD.

## Highlights

What is known, then list 2–4 bullet points.

1. Studies supporting the importance of vitamin D and omega-3 LCPUFA for brain function and structure, neurotransmitters and the glutamatergic system.
2. RCTs have investigated the effects of vitamin D and omega-3 LCPUFA each on core symptoms and problem behavior in patients with ASD. However, findings were mixed.

What is new, then list 2–4 bullet points.

1. This study analyses the effects of RCTs, focusing on the core symptoms of ASD, including changes in social functioning, behavioral functioning, language functioning and biomarkers, with research data from clinical that are more convincing.
2. Study found that the effects of omega-3 supplementation on ASD were too weak to conclude that core symptoms were alleviated. Vitamin D supplementation has a positive effect on behavioral functioning in ASD. Omega-3 and vitamin D combination supplementation had good combined effects in patients with ASD, with significant improvements in social and behavioral outcomes.

## Introduction

Autism spectrum disorders (ASD) are neurodevelopmental disorders that are characterized by impairments in social communication, interaction and two core symptoms such as repetitive, stereotyped behavior, narrow interests and activities (1). The prevalence of ASD has reached as high as 1.5% in developed countries (2, 3) and 0.7% among children aged 6–12 years in China (4). The exact etiology and pathogenesis of ASD remains unclear, and multisystem involvement and multiple co-morbidities often lead to an exacerbation of core symptoms in patients.

Given the unique disease manifestations and co-morbid conditions of ASD, the emergence of adaptive behavioral problems, language and communication problems, and emotional regulation problems will bring a strong stress shock to the parents of ASD, and the parents of ASD need to bear greater parenting pressure, life pressure, and economic treatment pressure than the parents of normal developmental children (5). The long-term accumulation of stress causes parents of ASD to have higher levels of depression and higher feelings of shame, fatigue, and powerlessness than parents of children with other developmental disorders (intellectual disability, attention deficit hyperactivity disorder) (6). Parents of ASD often show higher levels of psychological stress, caregiver stress, and parenting stress, and parents are prone to emotional changes such as anxiety and depression (7), physical changes such as sleep disorders, and even can lead to problems such as parental marital relationship breakdown, intergenerational conflict, and social isolation (8). Based on this, we desperately want to find a practical and effective method or drug that can improve the symptoms of ASD and reduce family stress. At the same time, we also try to be in the study of ASD mild patients, after treatment to restore social functioning how to better enter the regular school, whether a period of pre-training is needed before regular enrolment.

Essential fatty acids are a group of polyunsaturated fatty acids (PUFA) that can not synthesized by humans, including omega-3 and omega-6 and their derivatives. Omega-3 is an important component of phospholipids include linoleic acid (LA), alpha-linolenic acid (ALA), arachidonic acid (AA), eicosapentaenoic acid (EPA) and docosahexaenoic acid (DHA). Omega-3 plays an important role in the structure and function of cell membranes (9, 10). ALA is a precursor to omega-3 and can be converted to EPA and DHA. EPA and DHA are found in natural foods and are mainly supplemented through diet or deep-sea fish oil (11). DHA plays a role in cognitive function, neurotransmission, neuronal survival and attenuating neurodegeneration (12). The balance of essential fatty acids is essential for brain development and considered as a possible biomarker for ASD (13). Up to 60% of patients with ASD have some degree of immune dysfunction, suggesting a link between PUFA and inflammatory homeostasis (14). Reduced levels of omega-3 in the blood of ASD patients can lead to overproduction of the pro-inflammatory cytokine omega-6 (15). Low levels of omega-3 and omega-6 intake in ASD patients due to the fussy dietary behavior of individuals lead to increased levels of autoantibodies to neuronal and glial molecules and consequently to an omega-3/omega-6 ratio disorders (16). Lower omega-3 levels, or a disturbed omega-3/omega-6 ratio, increase inflammatory cytokines and oxidative stress, which in turn is associated with ASD symptoms (17).

Vitamin D is a neuroactive steroid that affects neuronal differentiation, axonal connections, brain structure and function. Exogenous and self-synthesized VitD3 undergoes two hydroxylation processes in the body before it exert biological effects. 25-hydroxyvitamin D3 [25 (OH)D3] is produced by the action of 25-hydroxylase (CYP2RA) in the microsomes and mitochondria of hepatocytes. 25(OH)D3 is released from the liver into the bloodstream and is the main stable form of vitamin D in the human blood circulation, so serum 25(OH)D3 levels are a marker of Vitamin D nutritional status. Studies have identified Vitamin D responsive elements in the promoter regions of numerous genes that regulate cell proliferation and differentiation, so-called 1,25 (OH) 2D3 target genes, and some genes can be directly affected by 1,25 (OH) 2D3, including p21 and p27. Studies have shown that 57% of children with ASD have vitamin D deficiency and another 30% have vitamin D insufficiency (18). Studies have shown that vitamin D supplementation has a beneficial effect on ASD symptoms (19, 20). In clinical studies, vitamin D supplementation has shown positive effects on autistic behavior, the effects of which may be due to vitamin D enhancing immune system function and reducing inflammation (21). Vitamin D plays an important role in the regulation of central and blood serotonin concentrations (22, 23). Mostafa and Al-Ayadhi study showed that vitamin D deficiency may be involved in the production of autoantibodies in autistic patients (24). Patrick and Ames showed that vitamin D deficiency may have a significant effect on serotonin, oxytocin and vasopressin concentrations in the brain (22).

Some randomized controlled studies have been conducted on the effects of vitamin D and omega-3 on the relief of core symptoms of ASD, with supporting the importance of vitamin D and omega-3 LCPUFA (EPA and DHA) for brain function and structure, neurotransmitters and the glutamatergic system, both of which have immunomodulatory, anti-inflammatory and antioxidant (25–27). Randomized controlled trials have investigated the effects of vitamin D and omega-3 LCPUFA each on core symptoms and problem behavior in patients with ASD. However, findings were mixed. Systematic evaluation itself can contain up-to-date knowledge and information with good reproducibility, economically, and can maximize the improvement of clinical medical practice and guide the direction of clinical research. The aim of this study was to explore the role of omega-3 and/or vitamin D on clinical symptoms in patients with ASD in an integrated manner by means of a systematic evaluation.

## Materials and methods

### Search criteria

Searching PubMed, Cochrane Library, Web of Science, EMBASE, CINAHL, Vip, CNKI, Wanfang, China Biomedical Database and other databases. The subject terms and search combinations are: omega-3 (“omega-3” OR “omega-3 PUFA” OR “ω-3”) AND vitamin D (“vitamin D” OR “1,25 dihydroxyvitamin d3” OR “d3,1,25 dihydroxyvitamin” OR “25 hydroxyvitamin d3”) AND/OR autism (“autism” OR “autism spectrum disorder” OR “ASD”). In addition to the database search, references to the identified studies were checked manually. Two subject members (Yu Jiang and Wenjun Dang) independently checked the title and abstract of each paper and filtered out irrelevant papers. Publication dates 2002–2022. A total of 1,002 papers were searched in English and 149 in Chinese.

### Search procedures

Literature screening criteria were set according to the PICO methodology: the participants (P), the interventions or exposure (I), the comparison (C), the outcome (O), the study design (S). P: Patients with ASD, diagnostic criteria: DSM-IV or DSM-5, or ADOS or ADI-R. I: Clinical studies of omega-3 or vitamin D or omega-3 + vitamin D supplementation in patients with ASD. C: omega-3 deficiency, or vitamin D deficiency. Outcome: ASD-related symptoms have improved. Study design: randomized controlled trial.

**Figure 1:**
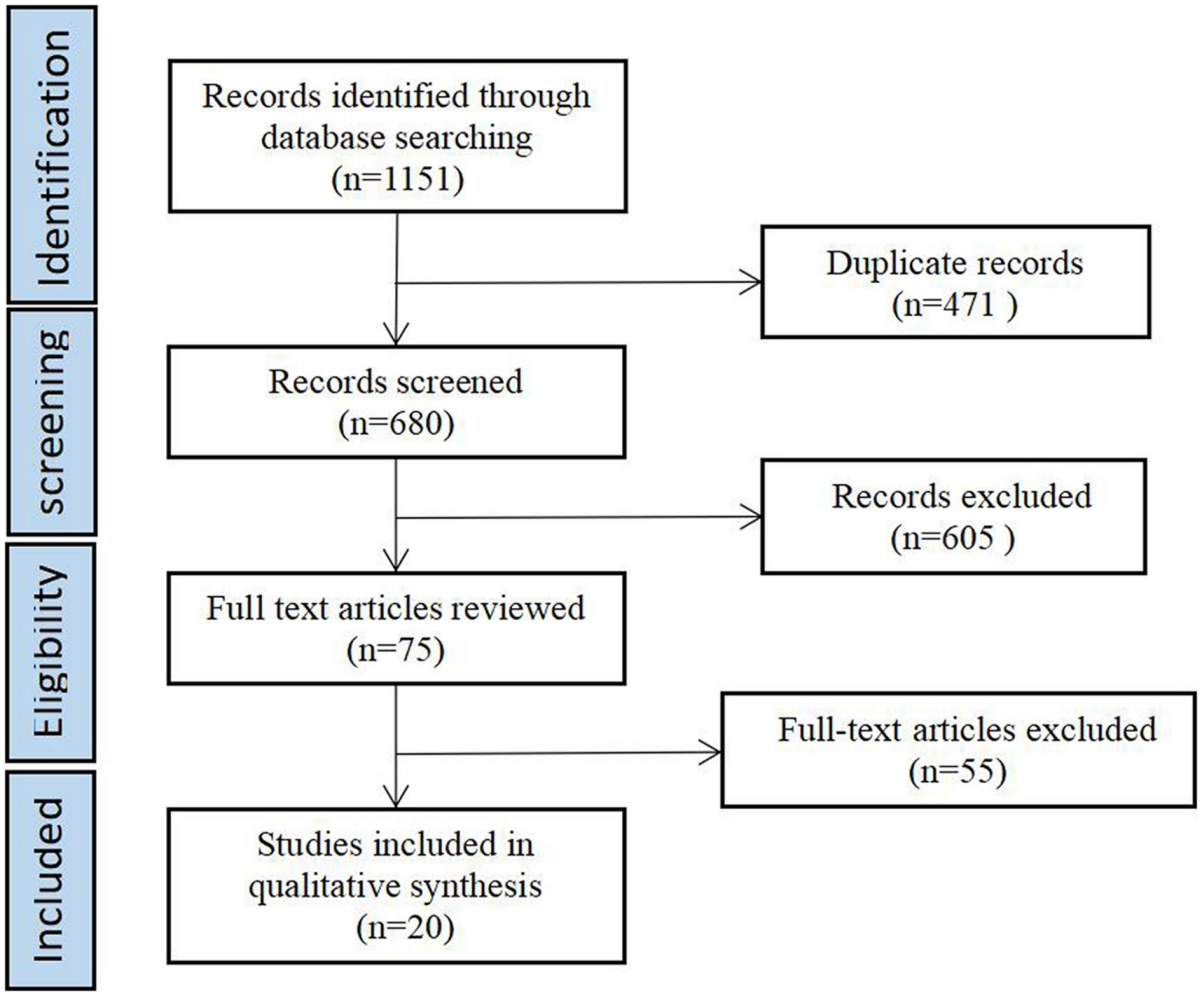
Flow diagram of paper selection.

The trial was divided into omega-3 control and observation groups that were well balanced and comparable between groups; or using their own pre- and post-control. Observations included at least one of the following items: stereotyped behavior, speech, social interaction, communication. Exclusion criteria: ①duplicate published literature; ②interventions influenced by other foods or medications so that the final treatment effect could not be judged. Eventually included 20 publications. For a flow chart of the literature screening, presented in Figure 1.

### Data analysis

Data were extracted independently by two reviewers from eligible studies and included the first author name, year, country, study design, sample size, age of participants, diagnostic criteria, vitamin D or omega-3 concentrations, intervention duration, and intervention outcomes.

### Quality appraisal and data extraction

Two investigators independently evaluated the quality of the selected literature, judged the inclusion and exclusion of literature, and ultimately obtained 20 literatures. The quality assessment of the included literature was based on the risk assessment scale developed by the Cochrane Collaboration Network for randomized controlled trials. The assessment consisted of seven entries. If each entry was low risk, the study was considered low risk and high quality; if one or more entries were of unknown risk, the study was considered unknown risk and moderate quality; if one entry was of high risk, the study was considered high risk and low quality. Two researchers exchanged checks with each other after assessment, and in case of disagreement, a third researcher was brought in to make a judgment. 20 RCTs were finally included, shown in Table 1.

**Table 1:** Quality assessment.

Study ID	Random sequence generation	Allocation concealment	Blinding of participants and personnel	Blinding of outcome data	Incomplete outcome data	Selective reporting	Other bias
Parellada et al. (28)	Low risk	Low risk	Low risk	Low risk	Low risk	Low risk	Unknow
Ooi et al. (29)	High risk	Unknow	High risk	Low risk	Low risk	Low risk	Unknow
Bent et al. (30)	Low risk	Low risk	Low risk	Low risk	Low risk	Low risk	Unknow
Bent et al. (31)	Low risk	Low risk	Low risk	Low risk	Low risk	Low risk	Unknow
Mankad et al. (32)	Low risk	Low risk	Unknow	Low risk	Low risk	Low risk	Unknow
Politi et al. (33)	High risk	High risk	Low risk	High risk	High risk	High risk	Unknow
Amminger et al. (34)	Unknow	Unknow	Low risk	Low risk	Low risk	Low risk	Unknow
Voigt et al. (35)	Low risk	Low risk	Low risk	Low risk	Low risk	Low risk	Unknow
Yui et al. (36)	Low risk	Low risk	Low risk	Low risk	Low risk	Low risk	Unknow
Doaei et al. (37)	Low risk	Low risk	Low risk	Low risk	Low risk	Low risk	Unknow
Javadfar et al. (38)	Low risk	Low risk	Low risk	Low risk	Low risk	Low risk	Unknow
Saad et al. (39)	Low risk	Low risk	Low risk	Low risk	Low risk	Low risk	Unknow
Saad et al. (20)	High risk	High risk	Low risk	Low risk	High risk	Low risk	Unknow
Duan (40)	High risk	High risk	Low risk	Low risk	High risk	High risk	Unknow
Kerley et al. (41)	Low risk	Low risk	Low risk	Low risk	Low risk	Low risk	Unknow
Feng et al. (42)	High risk	High risk	Low risk	Low risk	High risk	Low risk	Unknow
Mazahery et al. (43)	Low risk	Low risk	Low risk	Low risk	Low risk	Low risk	Unknow
Mazahery et al. (44)	Low risk	Low risk	Low risk	Low risk	Low risk	Low risk	Unknow
Mazahery et al. (45)	Low risk	Low risk	Low risk	Low risk	Low risk	Low risk	Unknow
Fang et al. (46)	Low risk	High risk	Low risk	Low risk	High risk	High risk	Unknow

The data were extracted independently by 2 researchers and exchanged for verification after extraction. The extraction included: basic information about each literature, including author, year, country, age, sample size, diagnostic criteria, intervention time, measurement tools, experimental reagents, and outcome indicators; extraction of data on the social, behavioral, and verbal treatment effects of patients with ASD in each literature.

## Results

### Summary of studies

A total of 20 papers were included all using randomized, double-blind, open clinical trial studies, sample size of 13–109, a total of 991 study subjects. Study come from 12 countries: USA, New Zealand, China, Egypt, Iran, Ireland, Austria, Canada, Japan, Switzerland, Spain, Italy. Three studies from USA, three from New Zealand, three from China, two from Egypt, two from Iran. All subjects were ASD patients, aged 2–40 years, and the duration of the study ranged from 6 weeks to 12 months, with omega-3 interventions lasting from 6 weeks to 6 months, vitamin D interventions lasting from 3 to 6 months, but the combined omega-3 and vitamin D supplementation trials all lasted 12 months. In the study design, the intervention studies for both omega-3 and vitamin D were two-group controlled studies and the combined omega-3 and vitamin D supplementation trial was a four-group controlled study with a reasonable study design.

Most of the included studies used the DSM scale to confirm the diagnosis of ASD patients (*n* = 19) and ADOS scale (*n* = 3), SCQ questionnaire (*n* = 3), and Wechsler Intelligence Scale (*n* = 2). Studies assessed social, behavioral and speech functioning in ASD, using the ABC scale (*n* = 12), SRS scale (*n* = 8), GARS scale (*n* = 7), CGI scale (*n* = 4) and BASC scale (*n* = 3). 12 studies reported changes in laboratory indicators, mainly considering the levels of EPA, DHA, 25(OH)D3, and related biochemical tests in the subjects. Specific results of the quality assessment of the literature are shown in Table 2.

**Table 2:** Summary of study details.

Intervention Type	Literature (country)	Age (years)	Sample size	Diagnostic criteria	intervention time	Measurement tool	experimental supplement		Biomarkers		Scale score	
							experimental group	Placebo group	experimental group	Placebo group	experimental group	Placebo group
Omega-3	Parellada et al. (28) Spain	5–17	67	Child psychiatrist，DSM-IV diagnosis of Pervasive Developmental Disorder	18 weeks (phase 1: 8 weeks; 2 weeks interva:effect removal time l;phase 2: 8 weeks)	①ω3 PUFAs ②TAS ③SRS ④CGI-S	Patients aged 5–11 years, 964.1 mg (EPA 577.5 mg + DHA 385 mg + Vitamin E 1.6 mg); Patients aged 12–17 years, 1157.01 mg (EPA 693 mg + DHA 462 mg + Vitamin E 2.01 mg)	Liquid paraffin and vitamin E at the same dose as the observation group	①AA/DHA intervention group(subject impact *F* = 8.248, *p* < 0.001) time×group effect(*F* = 11.548, *p* < 0.001) ②ω3/ω6(time×group effect *F* = 8.667,*p* < 0.001)	No improvement	No improvement	No improvement
Omega-3	Ooi et al. (29) Switzerland	7–18	41	Child psychiatrist [autistic symptoms rating at least moderate severity(CGI)], DSM-IV, WISC-IV, WPPSI	12 weeks	①SRS ②CBCL ③Blood status	15 mL liquid (Efamol Efalex) daily, 1 g/day of omega-3(DHA 840 mg,EPA 192 mg, pure evening primrose oil 1,278 mg)	No mentioned	①Percentage of AA:EPA (*p* = 0.0001) ②Percentage of omega-3 highly (*p* = 0.0001) ③Percentage of EPA (*p* = 0.001) ④Percentage of DHA (*p* = 0.0001)	No improvement	①SRS-Social awareness (*p* = 0.01) ②SRS-Social cognition (*p* = 0.0001) ③SRS-Social communication (*p* = 0.0001) ④SRS-Social motivation (*p* = 0.01) ⑤SRS-Autistic mannerisms (*p* = 0.0001) ⑥SRS—Total score (*p* = 0.0001) ⑦CBCL-Total score (*p* = 0.02) ⑧CBCL-Attention problems (*p* = 0.03)	No improvement
Omega-3	Bent et al. (30) USA	3–8	25	Expert clinician，ADOS、 SCQ > 12、DSM-IV	12 weeks	①ABC ②BASC ③CGI-I ④Free fatty acid changes	Orange-flavored pudding packets (EPA 350 mg and DHA 230 mg)	Orange-flavored pudding included safflower oil	①22:6n3 (DHA) (*p* = 0.02) ②20:5n3 (EPA) (*p* = 0.03) ③% Monounsaturated (*p* = 0.007) ④% Polyunsaturated (*p* = 0.04) ⑤% Omega-3 (*p* = 0.01) ⑥% Omega-9 (*p* = 0.01) ⑦cytokines TNF-α (*p* = 0.023)	No improvement	No improvement	No improvement
Omega-3	Bent et al. (31) USA	5–8	57	Professional personnel, ADOS, ADI-R, SCQ > 12	6 weeks	①ABC-H > 20 ②SRS ③CGI-I	Orange-flavored pudding packets (EPA 350 mg and DHA 230 mg)	Orange-flavored pudding included safflower oil	-	-	①ABC-Stereotypy Parent Ratings (*p* = 0.05) ②ABC-lethargy Parent Ratings (*p* = 0.01)	No improvement
Omega-3	Mankad et al. (32) Canada	2–5	38	No mentioned diagnostician, DSM-IV	6 months	①PDDBI ②BASC-2 ③PLS-4 ④CGI-I ⑤omega-3	First 2 weeks, EPA + DHA 0.75 g (1.875 mL once a day), after 2 weeks, the dose was doubled to 1.5 g (3.5 mL)	Placebo contained refined olive oil and medium chain triglycerides	No improvement	No improvement	No improvement	No improvement
Omega-3	Politi et al. (33) Italy	18–40	19	A doctor and a psychologist，DSM-IV、 WAIS	6 weeks	①The Rossago Behavioral Checklist (personal communication)	Two gelatin capsules of fish oil supplements containing 0.93 g of EPA and DHA	No mentioned	–	–	No improvement	No improvement
Omega-3	Amminger et al. (34) Austria	5–17	13	No mentioned diagnostician,DSM-IV	6 weeks	①ABC	1.5 g/d omega-3(EPA 0.84 g/d ，DHA 0.7 g/d)	Coconut oil 1 g(contained vitamin E 1 mg、fish oil 1 mg)	–	–	No improvement	No improvement
Omega-3	Voigt et al. (35) USA	3–10	48	An experienced clinician, DSM-IV, CARS≥30	6 months	①ABC ②CDI ③BASC ④TESS ⑤CGI-I	500 mg triglyceride oil capsules containing 200mg DHA daily	500 mg daily(250 mg corn oil and 250 mg soybean oil)500 mg daily(250 mg corn oil and 250 mg soybean oil)	–	–	①BASC Parent—social skills (*p* = 0.04) ②BASC Teacher—functional communication (*p* = 0.02)	No improvement
Omega-3	Yui et al. (36) Japan	6–28	13	2 independent psychiatrists，DSM-IV、WISC-IV > 80	16 weeks	①ABC ②SRS ③Biomarkers:PUFAs(DHA、ARA) ;Transferrin ; SOD	6 capsules a day(240 mg)containing ARA、DHA、0.16 mg astaxanthin	120 mg daily, Aravita containing olive oil capsule	Transferrin (*p* < 0.05)	No improvement	①ABC-Social withdrawal (*P* < 0.01) ②SRS-Communication (*p* < 0.05)	No improvement
Omega-3	Doaei,et al. (37) Iran	5–15	54	Clinician，ADOS、DSM-IV	8 weeks	①BMI ②FFQ ③GARS	1 g/d (180 mg EPA + 120 mg DHA) (Zahravi Company, Iran)	1 g/d (medium chain triglyceride)	–	–	①GARS-stereotyped behaviors (*p* = 0.02) ②GARS-social communication (*p* = 0.02) ③GARS-total score (*P* = 0.001)	No improvement
vitamin D	Javadfar et al. (38) Iran	3–13	52	Pediatrician，DSM-IV	15 weeks	①CARS ②ABC-C ③ATEC ④BMI ⑤Serum 25(OH)D3,IL-6, serotonin	300 IU/kg daily up to a maximum of 6,000 IU/d vitamin D syrup	No mentioned	25(OH)D3 (*p* = 0.006)	No improvement	①CARS total (*p* = 0.021) ②ATEC (*p* = 0.020)	No improvement
vitamin D	Saad et al. (39) Egypt	3–10	109	Two pediatricians, one psychiatrist, and two experienced psychologists; DSM-IV	4 months	①25(OH)D3 ②CARS ③ABC ④SRS ⑤ATEC ⑥Biomarkers:vitamin D levels, calcium, phosphorous, magnesium, glucose, potassium, alkaline phosphate, lead, blood urea nitrogen (BUN), serum creatinine, AST, ALT	300 IU/kg/d not to exceed 5,000 IU/day (Egyptian Ministry of Health)	Polysorbate 20	①Vitamin D levels (*p* < 0.001)	No improvement	①ABC—Irritability (*p* < 0.01) ②ABC—Hyperactivity (*P* = 0.014) ③ABC—Lethargy/social withdrawal (*p* = 0.003) ④ABC—Inappropriate speech (*p* < 0.01) ⑤ABC—Stereotypic behavior (*p* < 0.01) ⑥ATEC—Cognitive awareness (*p* < 0.05) ⑦ATEC—Behavior (*p* < 0.05) ⑧SRS—Social awareness (*p* < 0.001) ⑨SRS—Social cognition (*p* < 0.001) 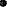 SRS—Autistic mannerism (*p* < 0.01) 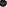 Total CARS scores (*p* = 0.02)	No improvement
Vitamin D	Saad et al. (20) Egypt	3–9	83	No mentioned diagnostician,DSM-IV	3 months	①CARS ②ABC	300 IU/kg/d not to exceed 5,000 IU/day (Egyptian Ministry of Health)	No mentioned	–	–	①ABC—irritability (*p* = 0.021) ②ABC—lethargy/social withdrawal (*p* = 0.028) ③ABC—hyperactivity (*p* = 0.01) ④ABC—stereotypic behavior (*P* = 0.04) ⑤CARS—Relating to people (*p* < 0.001) ⑥CARS—Emotional response (*p* < 0.001) ⑦CARS—Imitation (*p* < 0.001) ⑧CARS—Body use (*p* = 0.01) ⑨CARS—Object use (*p* = 0.01) 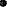 CARS—Adaptation to change (*p* = 0.004) 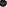 CARS—Listening response (*p* = 0.01) 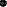 CARS—Visual response (*p* = 0.003) 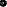 CARS—General impression (*p* < 0.001) 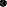 CARS—Total CARS score (*p* < 0.001)	No improvement
Vitamin D	Duan et al. (40) China	3–6	36	No mentioned, ICD-10, DSM-IV	3 months	①ABC ②CARS ③25(OH)D3	Alfacalcalcitol 400 IU/time, orally once a day; Vitamin D3 injection 150,000 IU/time, once a month intramuscular injection	No mentioned	①25(OH) D3 rised (*p* ≤ 0.001)	No improvement	①ABC—total score (*p* = 0.000) ; ②ABC—interaction (*p* = 0.002) ; ③ABC—somatic motor ability (*p* = 0.000) ; ④ABC—speech (*p* = 0.011) ; ⑤ABC—self-care individual scores (*p* = 0.000 ⑥CARS total score (*p* = 0.002)	No improvement
Vitamin D	Kerley et al. (41) Ireland	3–30	38	No mentioned, DSM-IV; SCQ > 15; aged <18 years	20 weeks	①ABC ②SRS ③DD-CGAS ④Biomarkers: complete blood count, 25(OH)D3 、 C reactive protein (CRP)	2000 IU/day	No mentioned	①25(OH) D rised (*p* = 0.0016)	No improvement	①DD-CGAS— self-care (*p* = 0.02)	No improvement
Vitamin D	Feng et al. (42) China	3–5	37	Pediatrician，DSM-IV	3 months	①ABC ②CARS ③ serum 25(OH)D3	150,000 IU per month, and orally 400 IU per day (in total 3 months)	No mentioned	①25(OH)D rised (*p* = 0.000)	No improvement	①total ABC scores (*p* < 0.05) ②ABC—sensory subscale (*p* < 0.05) ③ABC—social skills (*p* < 0.05) ④ABC—body and object use (*p* < 0.05) ⑤ABC—speech subscale (*p* < 0.05) ⑥ABC—social or self-help (*p* < 0.05) ⑦total CARS scores (*p* < 0.05)	No improvement
Omega-3 + vitamin D	Mazahery et al. (43) New Zealand	2.5–8	73	Pediatrician，DSM-IV	12 months	①ABC ②Biomarkers: full blood count, erythrocyte fatty acids, 25(OH)D3, calcium, albumin, iron studies (iron, iron binding capacity, ferritin, and transferrin saturation), vitamin B12, folate	Three groups -- VID group:vitamin D3(2000IU/day) ; OM group:omega-3 LCPUFA(722 DHA/day) ; VIDOM group:vitamin D3(2000IU/day)，DHA(722 mg/day)		No improvement	No improvement	①Irritability(VID vs. placebo (*p* = 0.01) OM vs. placebo (*p* = 0.001) VIDOM vs. placebo (*p* = 0.09)) ②Hyperactivity(VID vs. placebo (*p* = 0.047)) ③Lethargy(OM vs. placebo (*p* = 0.02))	No improvement
Omega-3 + vitamin D	Mazahery et al. (44) New Zealand	2.5–8	73	Pediatrician，DSM-IV	12 months	①SRS ②SPM ③Biomarkers: full blood count, erythrocyte fatty acids, and 25(OH)D3, calcium, albumin, iron studies (iron, iron binding capacity, ferritin, and transferrin saturation), vitamin B12, folate	Three groups -- VID group:vitamin D3(2000IU/day) ; OM group:omega-3 LCPUFA(722 DHA/day) ; VIDOM group:vitamin D3(2000IU/day)，DHA(722 mg/day)		No improvement	No improvement	①SRS-social awareness(OM and VIDOM) (*p* = 0.03) ②SRS-social communicative functioning(VIDOM) p < 0.1 ③SRS-total(OM) *p* < 0.1 ④SPM-taste/smell(VIDOM) p < 0.1 ⑤SPM-balance/motion (OM) p < 0.1	No improvement
Omega-3 + vitamin D	Mazahery et al. (45) New Zealand	2.5–8	67	Pediatrician，DSM-IV	12 months	①SRS ②Biomarkers: 25(OH)D3、RBC、IL-1β、calcium、albumin、iron、vitamin B12	Three groups -- VID group:vitamin D3(2000IU/day) ; OM group:omega-3 LCPUFA(722 DHA/day) ; VIDOM group:vitamin D3(2000IU/day),DHA(722 mg/day)		No improvement	No improvement	1.All children ①SRS-awareness (*p* = 0.01)——OM group ②SRS-awareness (*p* = 0.01)——VIDOM group ③SRS-social communicative functioning (*p* = 0.05)——VIDOM group 2.children with elevated IL-1β at baseline ①SRS-awareness (*p* = 0.01)——VID group ②SRS-awareness (*p* = 0.003)——OM group ③SRS-total (*p* = 0.01)——OM group ④SRS-social communicative functioning (*p* = 0.03)——OM group ⑤SRS-motivation (*p* = 0.05)——OM group ⑥SRS-awareness (*p* = 0.01)——VIDOM group ⑦SRS-social communicative functioning (*p* = 0.05)——VIDOM group	
Omega-3 + vitamin D	Fang et al. (46) China	5–12	48	No mentioned,DSM-IV	12 months	1.CARS	Three groups——VD group:VD 800 U/d ; ω3 group:ω3 900 mg/d ; Combination group:VD 800 U/d + ω3 900 mg/d		–	–	①VD group and Combination group(compared to placebo group):emotional response, imitation, relationship with inanimate objects, adaptation to environmental change, proximity sensory response, visual response, anxiety response, and overall impression score decreased (*p* < 0.05) ②ω3 group and placebo group:auditory response, visual response, anxiety response score decreased (*p* < 0.05) ③Combination group and ω3 group:Emotional response, imitation, relationshi*p* with inanimate objects, adaptation to environmental change, anxiety response decreased (*p* < 0.05) ④Combination group and VD group:Visual response, auditory response (*p* < 0.05)	No improvement

①DSM-IV, Diagnostic and Statistical Manual of Mental Disorders ; ②TAS, Otal Antioxidant Status; ③SRS, Social Responsiveness Scale; ④CGI, Clinical Global Impression; ⑤WISC-IV, Wechsler Intelligence Scale for Children; ⑥WPPSI, The Wechsler Preschool and Primary Scaleof Intelligence; ⑦CBCL, Child Behavior Check List; ⑧ADOS, The Autism Diagnostic Observation Schedule; ⑨SCQ, Social Communication Questionnaire; 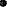
BASC, Behavior Assessment System for Children; 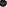
ADI, Autism Diagnostic Interview; 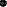
ABC, Aberrant Behavior Checklist; 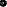
PDDBI, PDD Behavior Inventory; 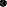
PLS, Preschool Language Scale; 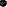
WAIS, Wechsler Adult Intelligence Scale; 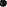
CDI, Child Development Inventory; 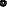
TESS, Treatment Emergent Symptom Scale; 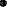
BMI, Body Mass Index; 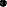
FFQ, Food Frequency Questionnaire; 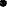
GARS, Gilliam Autism Rating Scale; 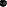
DD-CGAS, The Developmental Disabilities — Children’s Global Assessment Scale; 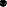
SPM, Sensory Processing Measure.

### ASD core symptoms

All 20 papers examined the effects of omega-3 and/or vitamin D on core symptoms of ASD, including social function, behavioral function, and speech function.

### Social functioning

Eight papers examined the effects of omega-3 on social function in people with ASD, mainly using the SRS scale, including social awareness, social cognition, social communicative functioning, social motivation, autistic mannerism.

Four of the omega-3 intervention studies used the SRS scale. Ooi (29) study showed that after 12 weeks of omega-3 intervention, for subjects assessed using the SRS scale, there were significant improvements in social perception, social cognition, social communication, social motivation and autistic behavioral styles, and total scores. Yui (36) showed significant improvements in social communication after 16 weeks of omega-3 supplementation. Parellada (28) showed no improvement in overall social functioning after omega-3 supplementation, but it was also noted in the study that Parellada used a crossover design and that there could be a sequential effect during the treatment phase, which would confound the treatment effect, Although the researchers designed an effect removal time of 2 weeks to avoid sequential effects, the *in vivo* metabolic cycle of omega-3 was not further clarified to ensure that the elution period was sufficiently long. Bent (31) and others showed no improvement in social functioning after 6 weeks of omega-3 supplementation.

Vitamin D intervention study had two studies using the SRS scale. Saad (39) showed that after 4 months of vitamin D supplementation, the SRS-autistic mannerism, SRS-social cognition, and SRS-social awareness statistical studies have significantly improved. Kerley (41) showed that after 20 weeks of supplementation, there was no difference in the individual data in the SRS scale.

In contrast, in the combined omega-3 and vitamin D intervention studies, two studies used the SRS scale and both were with the same group of researchers. Mazahery (44, 45) showed that after 12 months of supplementation, the SRS-social awareness, SRS-social communicative functioning, and SRS-total were statistically significant before and after supplementation.

In a comprehensive analysis, ten supplementary studies involving omega-3, four studies used the SRS scale but only two showed statistical differences, but of these (29) studies had two high risk and two unknown risk, with low quality literature. In the other study (36), only one social communicative functioning showed a statistical difference. Of the six supplementation studies involving Vitamin D, two used the SRS scale, but only one showed statistical differences, which only three indicators also showed statistical differences. However, 4 studies with combined omega-3 and vitamin D interventions, only two used the SRS scale, with all three indicators showing a difference.

### Behavioral functioning

To evaluate behavioral functioning, the ABC scale, the BASC system, the CBCL and GARS were used.

### Aberrant behavior checklist

Twelve papers used the ABC scale, examined the effects of omega-3 on abnormal behavior in people with ASD, which consists of five factors: irritability, hyperactivity, lethargy/social, inappropriate speech, stereotypic behavior. Bent (31) showed that taking omega-3 for six weeks had a significant improvement in lethargy, stereotypic behavior in the parent-rated version of the ABC scale for subjects in 2014. Bent (30) showed no effect on abnormal behavior in subjects by taking omega-3 for 12 weeks in 2011. Stephen Bent conducted two controlled clinical studies of omega-3 in patients with ASD in 2011 and 2014, respectively, and the observation group in the 2011 study showed no difference compared to the control group. There was no difference in the ABC scale. The reasons for this analysis were considered to be possibly due to the inclusion of too few patients (*n* = 25) to determine efficacy. Yui (36) showed that after 16 weeks of omega-3 supplement, there was a significant improvement in social withdrawal in the observation group compared to the control group, with a significant difference. Amminger (34) showed that after six weeks of omega-3 supplement, a repeated measures ANOVA showed no significant difference between the observation group and the control group, but there was a tendency for the observation group to have remission of hyperactivity symptoms. Voigt (35) showed that after 6 months of omega-3 supplementation, there was no significant difference between the observation group and control group were not significantly different.

Saad (39) study showed a significant improvement in ABC scores in the vitamin D supplementation group compared to the placebo group. There were significant changes in irritability, hyperactivity, social withdrawal, stereotypic behavior, and inappropriate speech statistics. Furthermore, this study found a high correlation between serum 25 (OH)D3 levels and ABC-total scores and ABC-language subscale scores, with significant reduction in ABC after vitamin D supplementation, and this treatment effect was more pronounced in younger patients. Duan (40) showed a statistically significant reduction in the total ABC score and interaction, somatic motor ability, speech and self-care individual scores for before and after 3 months of treatment in the study compared to the pre-treatment period. And further divided into early treatment group (age ≤ 3 years) and late treatment group (age > 3 years), for the difference in total ABC score early treatment group was greater than late treatment group, the difference was statistically significant. Moreover, this study also showed that serum 25(OH)D3 levels were negatively correlated with total ABC scores and individual ABC verbal ability scores. However, further statistical analysis showed that serum 25(OH)D3 levels did not correlate with the gender. Considering further, serum 25(OH)D3 levels had a significant negative correlation with anti-MAG, and anti-MAG deficiency may affect anti-MAG levels, suggesting that anti-MAG is highly correlated with ASD, serum 25(OH)D3 levels are correlated with the degree of behavioral abnormalities in ASD patients (47). Feng (42) monthly intramuscular vitamin D3 injections (150,000 IU) and daily oral vitamin D3 (400 IU) had statistically significant differences in total ABC scores, social skills, body and object use, speech, and social or self-help compared before and after treatment. Their study also showed that 25(OH)D3 levels were negatively correlated with total ABC scores and speech scores on the ABC. Saad (20) showed statistically significant improvements in irritability, lethargy/social withdrawal, hyperactivity and stereotypic behavior on the ABC scale before and after vitamin treatment. Kerley (41) showed that there was no statistically significant difference in ABC levels for patients after 20 weeks of vitamin D supplementation, but the original article also speculated that the lack of change in ABC levels may be related to the milder symptoms of the included ASD patients. Javadfar (38) showed that after 15 weeks of vitamin D supplementation, there was no statistically significant effect on the pre- and post-ABC scale symptom statistics.

Mazahery (43) showed that combined omega-3 and vitamin D supplementation showed statistical differences in irritability, hyperactivity, and lethargy in ABC.

In the comprehensive analysis, five of the ten omega-3 supplementary studies used the ABC scale, but only two of them showed statistical differences for a total of three subscales, so the effect was not significant, vitamin D supplementation had a greater impact on patients’ behavioral functioning. In six studies of Vitamin D supplementation, all of which used the ABC scale to investigate the impact on patients’ behavioral functioning, four studies showed that patients’ behavioral functioning improved in various ways after supplementation, with statistically differences. However, it should also be noted that of the Vitamin D supplementation studies, Saad K’s (20) study had three high risk and one unknown risk; Xiaoyan’s (40) study had 4 high risk and 1 unknown risk; and Feng J’s (42) study had 3 high risk and 1 unknown risk, with low quality literature. However, 4 studies with combined omega-3 and vitamin D interventions, only 1 used the ABC scale and showed statistical differences in some of the indicators.

### Behavior assessment system for children

Three articles used the BASC system to evaluate children’s behavior, all of which were also omega-3 supplementation studies. The BASC system is divided into self-reported, parent-rated, teacher-rated, and student-observed versions, with the aim of providing a comprehensive assessment of children’s behavior using different evaluators. Voigt (35) used teacher-rated and parent-rated, and after 6 months of the omega-3 intervention, the observation group had significant differences in the social function and the communication function compared to the control group. Mankad (32) used the parent-rated versions in their study after 6 months, monitored at baseline, week 12 and week 24, but there was no significant difference between the observation and control groups. Bent (30) did not specify which version of the BASC system was used, but there was no significant difference before and after the intervention.

### Child behavior check list

The CBCL is used to assess children’s social skills and behavioral problems in omega-3 supplemental study. Ooi’s (29) study used a parent-reported version and showed a significant difference between the results of the observation groups and control groups, improvement in social and attention problems for patients.

### Gilliam autism rating scale

Seven papers used the GARS scale, which is a standardized tool for assessing autism spectrum disorders and other severe behavioral disorders.

Saeid Doaei (37) showed an improvement in GARS stereotypical behavior, social communication, and total scores in the observation group after an 8-week omega-3 intervention, with significant differences.

The GARS scale was used in all 6 vitamin D supplementation studies. Javadfar (38) showed that the decrease in total GARS scores before and after the intervention, was significantly greater than in the placebo group, and the difference was statistically significant. Khaled Saad (39) showed a significant improvement in the total CARS score in the vitamin D supplementation group compared to the placebo group after a study period of 4 months. Khaled Saad (20) study shown that GARS scale (relating to people, emotional response, imitation, body use, object use, adaptation to change, listening response visual response, general impression, total CARS score), before and after treatment the difference was statistically significant. Serum 25(OH)D3 levels were significantly and negatively correlated with CARS scores. Children with 25(OH)D3 levels of >40 ng/mL all had improved CARS scores. Xiaoyan (40) showed a statistically significant reduction in total CARS score after 3 months of treatment compared to pre-treatment. Feng (42) showed a statistically significant difference in the reduction in total CARS score between the early treatment group and the late treatment group. However, in their study, CARS scores did not correlate with 25(OH)D3 levels.

Fang’s study (46) showed that children in the VD group and the combination group had significantly lower (*p* < 0.05) scores for emotional reactions, imitation (words and actions), relationship with inanimate objects, adaptation to environmental changes, proximal sensory reactions, visual reactions, anxiety reactions, and overall impressions, suggesting that taking VD or VD combined with omega-3 was effective in improving the above symptoms in children with ASD. Compared to the placebo group, children in the omega-3 group had significantly lower auditory response, visual response, and anxiety response scores only (*p* < 0.05). Compared to the omega-3 group, children in the combination group showed significantly lower scores for emotional responses, imitation (words and actions), relationship to inanimate objects, adaptation to environmental changes, and anxiety responses (*p* < 0.05). The combined medication group was more effective in improving the children’s visual and auditory responses than the VD group (p < 0.05).

In the comprehensive analysis, ten omega-3 supplementary studies,only one used the GARS scale, but only three indicators showed statistical differences. Six vitamin D supplementation studies, all of which used the GARS scale, there was a significant increase in the total GARS score in each of these studies, while a total of 10 indicators showed statistical differences in the study (20). However, four studies with combined omega-3 and vitamin D interventions, only one used the GARS scale and showed statistical differences in a number of indicators.

### Clinical global impression

Five articles used the CGI Scale, all of which were omega-3 supplemented studies, in which the CGI-I scale was considered to be the commonly used measure in all clinical trials of patients with ASD to evaluate clinical outcomes, but all of their results showed no significant differences before and after the intervention.

### Speech function

Mankad (32) used the PLS (Preschool Language Scale) to assess the language skills at the beginning and at 24 weeks. However, the results of the study showed that there was no significant difference between the observation group and the control group. Mankad’s language assessment was conducted at the beginning and at the end of the study, using linear regression in the statistics, and lacked a follow-up phase during the study.

### Biomarkers changes

Specific blood fatty acid levels have been associated with changes in the core symptoms of ASD (48). Related studies have shown that red blood cells and brain tissue have different polyunsaturated fatty acid compositions. Polyunsaturated fatty acids are more readily available in erythrocyte membranes than in brain tissue, and tests targeting polyunsaturated fatty acids in blood can help to suggest the amount of polyunsaturated fatty acids in brain tissue.

Parellada (28) examined the ratio of omega-3 in erythrocyte membranes (AA/DHA, AA/EPA, ω3/ω6) and plasma total antioxidant status (TAS). The results of the study showed a significant time effect and a time group effect with significant differences in the AA/DHA and ω3/ω6 ratios between the two groups. Ooi (29) study considered the blood levels of patients and tested AA/EPA, omega-3, EPA, and DHA levels. After the intervention, there was a significant decrease in the percentage of AA/EPA and a significant difference in the difference in omega-3, EPA, and DHA changes, and the study showed that changes in blood level indicators were associated with a decrease in the severity of ASD behaviors. In the Bent (30) study, omega-3 percentages increased in the intervention group and decreased slightly in the control group. DHA and EPA levels increased in the intervention group compared to the control group and the difference was significant. Nine individual fatty acids (15:0, 22:0, 24:0, 18:1n9, 22:4n6, 22:5n6, 20:5n3 (EPA), 22:5n3, 22:6n3 (DHA)) and four of the eight category percentages (% monounsaturated fatty acids, % polyunsaturated fatty acids, % omega-3, omega-9) also showed significant differences in change over the course of the study, indicating that 12 weeks of omega-3 supplementation significantly affected fatty acid distribution. Stephen Bent also measured 29 cytokines, but only one (TNFa) showed a significant difference in mean change over the course of the study between groups, with TNFa increasing in the observation group and decreasing in the control group. Yui (36) examined plasma levels of SOD, Transferrin and PUFAs and there was a trend toward a significant difference in the change in plasma Transferrin levels between the two groups (*p* = 0.03) ，and a trend toward a significant difference in plasma SOD levels (*p* = 0.08), and plasma DHA (*p* = 0.74) and ARA (*p* = 0.86) levels were not statistically significant. Transferrin is involved in signal transduction, SOD plays a role in lipid signaling in defense against oxidative stress, and the interrelationship between SOD and transferrin mediates the signaling pathway. Mankad (32) examined omega-3 fatty acid levels and cytokines in his study, but the differences were not significant between groups.

In Javadfar’s (38) study, Serum 25(OH)D3, IL-6, serotonin levels were measured in patients, but only serum 25(OH)D3 levels increased significantly after vitamin D supplementation, with a statistically significant difference (*p* = 0.006). IL-6 is an indicator of chronic inflammation and an independent and reliable predictor of ASD, but no differential change was found in this study. After the study (39), serum 25(OH)D3 levels were significantly higher in the observation group, with a statistically significant difference (*p* = <0.001), and no differences in any other biomarkers. Serum 25(OH)D3 levels were significantly higher in the observation group before and after treatment, and the difference was statistically significant (*p* = 0.000) (40). Serum 25(OH)D3 levels were significantly higher before and after supplementation (41), with a statistically significant difference (*p* = 0.0016), while there were no differences in other biochemical indicators. Serum 25(OH)D3 levels were significantly higher in all patients after vitamin D supplementation (*p* = 0.000) (42).

Study (44) showed that analysis of serum 25(OH)D3 concentration and omega-3 index showed a significant (*p* < 0.01) interaction between follow-up time and treatment time point and observation group. The study (43) showed no statistically significant difference in changes in serum 25(OH)D3 concentrations or omega-3 index before and after supplementation. Studies (45) have shown no statistical difference in changes in serum 25(OH)D3 concentrations or omega-3 index before and after supplementation.

In a comprehensive analysis, thirteen of the twenty papers dealt with relevant biochemical indicators. Ten studies of omega-3 supplementation included in this study, five considered biomarkers at the time of testing, with changes in blood levels of fatty acids. Four of these studies showed a significant difference in blood levels between the observation group and the control group after omega-3 supplementation, with significant differences. Of the 6 Vitamin D supplementation studies, 5 considered biomarkers at the time of testing and serum 25(OH)D3 concentrations were increased in five of these studies, with a statistically significant difference between before and after. However, of the four studies of combined omega-3 and vitamin D interventions, three considered biomarkers and only one (44) showed statistical differences in omega-3, serum 25(OH)D3 concentrations.

## Discussion

Given that there are no effective drugs for the treatment of ASD (49), and considering the food selectivity of patients with ASD, some studies are gradually looking at nutritional therapy as a way to compensate for nutritional deficiencies and alleviate core symptoms (50), based on which fatty acid supplementation stands out among the many nutritional therapies (51). Ten studies analyzing the effects of omega-3 supplementation in people with ASD showed variation in the results of the literature. Only some of the studies showed that supplementation was effective in improving the core symptoms associated with ASD, with the best study being that of Ooi. However, the Ooi study was relatively low quality, with two high risk assessments and two unknown risk assessments, and the quality of the literature was low. Six studies analyzed the effects of vitamin D supplementation in patients with ASD, vitamin D supplementation improved some of the core symptoms of ASD in patients with ASD, mainly behavioral functioning. However, the results of the literature included in this study were slightly mixed. Four studies analyzed the effects of combined omega-3 and vitamin D supplementation in patients with ASD, and the combined effect was good, with significant improvements in social and behavioral outcomes.

Adherence to nutritional interventions is an important factor influencing the effectiveness of the study. Studies have shown that high adherence with family interventions for ASD facilitates treatment delivery, helps relieve patients’ symptoms and improves their intelligence (52). Patient adherence control does have a direct impact on the effectiveness of studies of omega-3 supplementation in patients with ASD. The literature included in this study also varied in the approach taken to adherence control. Parellada (28) monitored patient adherence in a number of ways, with the team asking participants to hand in all omega-3 capsules (empty or not), along with a weekly calendar, and the researcher would also count the number of medications. There was also a researcher who conducted telephone assessments every other week during the intervention to check patient adherence and adverse events. Bent (30) was contacted by telephone at week 2, week 8, a brief assessment at week 6 and a final visit at week 12 during. Bent (31) used an internet-based random controlled trial approach in his 2014 trial, in which 863 registered members were invited via email upfront, and interested parents were invited to complete an initial test via an embedded link in an email to a screening questionnaire. A parent or carer or teacher is also required who is willing to complete the baseline information and assessment via email. Parents of children screened through eligibility, online informed consent is completed in the form of an electronic signature. And all participants in the study have the option to speak to the researcher by telephone before signing the informed consent form. Parents participating in the study then received weekly follow-up emails reporting medication adherence, medical problems and were also assessed by email at week 3 and week 6. The study collected data in a manner consistent with the US Food and Drug Administration (FDA) and regulations in the Health Insurance Portability and Accountability Act. Once a subject has had an adverse event and it is entered into the web-based platform, the two principal investigators receive an email alert from the platform, will consult on the adverse event and call the parents to record further information about it. This internet-based experimental approach has shown the advantages of low cost, rapid registration, high completion rate and ease of participation, and facilitates the replication of more studies at a later stage.

Patients with ASD face their own symptoms of stereotypical behavior, communication disorders, social interaction disorders, and the long duration of the disease, the high cost of treatment, the burden on families and the psychological burden on carers. These factors have a significant impact on adherence with ASD, and more ways to increase patient compliance management can also help patients recover their social functioning. It was also found during the study that Bent (30) lost one patient in the 2011 study but none in the 2014 study. It can also be seen that an internet-based, multi-path intervention approach can increase patient adherence. Current expert consensus and guidelines for the management of patients with ASD mention the need for long-term interventions for patients with ASD (53–55), and adherence to treatment is an important indicator in intervention studies and an important basis for ensuring the effectiveness of long-term treatment for patients with ASD (56).

Interleukin-1β is frequently elevated in the plasma of children and adults with ASD (57). Mutations and polymorphisms in IL-1β and its receptor have been shown to be associated with ASD and cognitive performance (58, 59). The findings suggest that participants with higher levels of inflammation are immune responders if the intervention itself is immunomodulatory, whereas participants with higher levels of inflammation are not inflammation-responsive if the intervention has no immunomodulatory effect. Studies have shown immune alterations in the cerebrospinal fluid and peripheral blood of patients with ASD (57), with associated elevations of pro-inflammatory cytokines such as IL-1α and β, IL-1Ra, IL-4, IL-6, IL-10, TNF-α, and IFN-γ (60). On this basis it is possible to speculate that omega-3 and vitamin D may improve the clinical symptoms of ASD through the inflammatory response (61). Both studies (44, 45) come from the same group of researchers, but the study (45) discusses more profoundly the alteration of pretreatment inflammatory status on the therapeutic effects of combined vitamin D and omega-3 interventions (45). Baseline information on participants’ inflammatory status (IL-1ra, IL-6, and hs-CRP) was included prior to the study and stratification of inflammatory status was performed prior to the start of the study, with results showing that participants with high inflammatory status showed more improvement in treatment outcomes than the placebo group. Moreover, in the study (45), there were no differences in IL-1β statistics between the four groups at baseline, but a trend toward greater improvement in SRS-total, SRS-social communicative functioning, and SRS-RRB occurred when IL-1β was elevated over the course of the study.

It should also be noted that omega-3 supplementation does have associated adverse effects, with Parellada (28) showing that the only significant adverse event during the intervention was a small increase in total cholesterol during the trial. In the Bent (30) study, five subjects in the observation group reported adverse events: 2 rashes, 1 upper respiratory tract infection, 1 nosebleed and 1 exacerbation of gastrointestinal symptoms. However, there were also 4 adverse events in the control group, 3 increases in hyperactivity and 1 increase in self-stimulatory behavior. The difference between the observation and control groups was not significant in comparison. Amminger (34) showed that in the observation group mild adverse events occurred as fever, but the control group also had headache and insomnia. In addition to this there were associated adverse events, all of which were mild, but the difference between the observation and control groups was not significant. Of concern is the predominance of adverse events such as gastrointestinal problems (diarrhea, vomiting) (30, 31, 44, 46). Vitamin D supplementation studies have been associated with some adverse reactions. In the study by Javadfar (38) there were 3 patients who discontinued treatment: 2 due to rash and 1 due to diarrhea. Five patients in Khaled Saad’s study (39) presented with rash, pruritus and diarrhea. In the study by Mazahery (44), a rash, facial papules, and red ears were seen.

We sorted out the criteria for definition of vitamin D and supplementation criteria by the society and related organizations further in the last decade 2013–2023. According to Table 3, it can be seen that there are different definition criteria regarding vitamin D. There is no exact international standard definition and there are no precise criteria for vitamin D supplementation, but further analysis shows that the existing criteria for supplementation in children tend to apply the criteria of 0-6 months: 400 IU/day, 6–12 months: 400–600 IU/day, but it is important to note that the proposed criteria for supplementation are not In the context of ASD disease, it is accurate to say that there is no precise international standard for the amount of supplementation needed for children with ASD. And with further reference to Table 2, there is no complete harmonization of supplementation levels for children with ASD.

**Table 3:** Definition of vitamin D status and supplementation criteria.

NO	Title	Year	Society/Organization	Diagnostic standards					Supplementary standards
				Severe deficiency	Deficiency	Insufficiency	Sufficiency	toxicity	
1	Guidelines for Preventing and Treating Vitamin D Deficiency: A 2023 Update in Poland (62)	2023	Nutrition-related expert groups	-	<20 ng/mL	20–30 ng/mL	30-50 ng/mL	-	[1] 0–6 months: 400 IU/day; [2] 6–12 months: 400–600 IU/day; [3] 1–3 years: 600 IU/day; [4] 4–10 years: 600–1000 IU/day; [5] Adolescents (11–18 Years): 1000–2000 IU/day; [6] Adults (19–65 Years):1000–2000 IU/day; [7] Younger Seniors (>65–75 Years): 1000–2000 IU/day; [8] Older Seniors (>75–89 Years) and the Oldest Old Seniors (90 Years and Older): 2000–4,000 IU/day; [9] Pregnancy and Lactation: 2000 IU/day
2	Definition,Assessment, and Management of Vitamin D Inadequacy: Suggestions, Recommendations, and Warnings from the Italian Society for Osteoporosis, Mineral Metabolism and Bone Diseases (63)	2022	Italian Society for Osteoporosis, Mineral Metabolism and Bone Diseases	–	<10 ng/mL	<20 ng/mL	20-50 *n*g/mL	-	No mention
3	Vitamin D and calcium intakes in general pediatric populations: A French expert consensus paper (64)	2022	Group of Experts in Paediatric Related Medicine	<10 ng/mL	<20 ng/mL	20–29 ng/mL	30-60 ng/mL	>80 ng/mL	[1] 0–18 years: 400 IU - 800 IU vitD/ day; [2] 2–18 years: intermittent supplementation in the case of non adherence, vitD3 with either 50,000 IU quarterly or 80,000–100,000 IU twice in fall and winter.
4	Clinical Practice in the Prevention, Diagnosis and Treatment of Vitamin D Deficiency: A Central and Eastern European Expert Consensus Statement (65)	2022	Group of Experts in Paediatric Related Medicine	-	<20 ng/mL	20–30 ng/mL	30-50 ng/mL	>100 ng/mL	[1] Healthy adults: 800–2000 IU/day; [2] Elderly(>65 years): 800–2000 IU/day; [3] Hospitalized/institutionalized individuals: 800–2000 IU/day;
5	Indian Academy of Pediatrics Revised (2021) Guidelines on Prevention and Treatment of Vitamin D Deficiency and Rickets (66)	2021	Indian Academy of Pediatrics	-	<12 ng/mL	12–20 ng/mL	>20 ng/mL	-	[1] Infancy:400 IU/day; [2] Childhood:400 IU/day; [3] Adolescents:600 IU/day;
6	Vitamin D testing (67)	2019	Clinical Practice Guidelines and Protocols in British Columbia	-	12 ng/mL	12–20 ng/mL	≥20 ng/mL	>50 ng/mL	[1] Infants 0–6 months: 400 IU (10 μg)/D allowance;1,000 IU (25 μg)/D tolerable upper intake level; [2] Infants 7–12 months: 400 IU (10 μg)/D allowance;1,500 IU (38 μg)/D tolerable upper intake level; [3] Children 1–3 years: 600 IU (15 μg)/D allowance; 2,500 IU (63 μg)/D tolerable upper intake level; [4] Children 4–8 years: 600 IU (15 μg)/D allowance; 3,000 IU (75 μg)/D tolerable upper intake level; [5] Children and adults 9–70 years (including pregnant and lactating women): 600 IU(15 μg)/D allowance; 4,000 IU(100 μg)/D tolerable upper intake level; [6] Adults >70 years: 800 IU (20 μg)/D allowance; 4,000 IU (100 μg)/D tolerable upper intake level
7	Vitamin D in pediatric age: consensus of the Italian Pediatric Society and the Italian Society of Preventive and Social Pediatrics,jointly with the Italian Federation of Pediatricians (68)	2018	Italian Pediatric Society and the Italian Society of Preventive and Social Pediatrics	<10 ng/mL	<20 ng/mL	20–29 ng/mL	≥30 ng/mL	–	[1] 0–12 months (1) Infants without risk factors: 400 IU/day (2) Infants with risk factors: 1000 IU/day [2] 1–18 years (1) 600–1,000 IU/day
8	Vitamin D Supplementation Guidelines for General Population and Groups at Risk of Vitamin D Deficiency in Poland —Recommendations of the Polish Society of Pediatric Endocrinology and Diabetes and the Expert Panel with Participation of National Specialist Consultants and Representatives of Scientific Societies—2018 Update (69)	2018	Polish Society of Pediatric Endocrinology and Diabetes and the Expert Panel with Participation of National Specialist Consultants and Representatives of Scientific Societies	<10 ng/mL	10-20 ng/mL	20–30 ng/mL	30-50 ng/mL	>100 ng/mL	[1] 0-6 months:400 IU/day; [2] 6–12 months: 400–600 IU/day; [3] 2–10 years: 600–1,000 IU/day; [4] 11–18 years: 800–2000 IU/day; [5] >18 years: 800–2000 IU/day; [6] >75 years: 2000–4,000 IU/day; [7] Pregnancy and lactation:2000 IU/day;
9	Assessment criteria for vitamin D deficiency/ insufficiency in Japan—proposal by an expert panel supported by Research Program of Intractable Diseases, Ministry of Health, Labour and Welfare, Japan, The Japanese Society for Bone and Mineral Research and The Japan Endocrine Society (70)	2017	An expert panel supported by Research Program of Intractable Diseases, Ministry of Health, Labour and Welfare	-	<20 ng/mL	20–30 ng/mL	≥30 ng/mL	-	No mention
10	Assessment criteria for vitamin D deficiency/ insufficiency in Japan: proposal by an expert panel supported by the Research Program of Intractable Diseases, Ministry of Health, Labour and Welfare, Japan, the Japanese Society for Bone and Mineral Research and the Japan Endocrine Society (70)	2017	Japanese Society for Bone and Mineral Research, Japan Endocrine Society	-	<20 ng/mL	20–29 ng/mL	≥30 ng/mL	-	No mention
11	Clinical practice guidelines for vitamin D in the United Arab Emirates (71)	2016	United Arab Emirates	-	<20 ng/mL	20–29 ng/mL	≥30 ng/mL	-	[1] Breastfed infants: 400 IU/day up to age 6 months, 400–600 IU/day between 6 and 12 months; [2] Children and adolescents of age 1–18 years:600–1,000 IU/day; [3] > 18 years: 1000–2000 IU/day; [4] The elderly (over 65 years): 2000 IU/day; [5] Pregnant and breast feed women: 2000 IU/day; [6] Premature infants: 400–800 IU/day; [7] Obese, individuals and those with metabolic syndrome: 2000 IU/day; [8] Individuals with dark skin complexions and for night workers: 1000–2000 IU/day;
12	Global Consensus Recommendations on Prevention and Management of Nutritional Rickets (72)	2016	Global Consensus for rickets	-	<12 ng/mL	12–19 ng/mL	≥20 ng/mL	>100 ng/mL	The supplemental quantities mentioned in the article are all for nutritional rickets and osteomalacia
13	Vitamin D and bone health: a practical clinical guideline for management in children and young people (73)	2015	National Osteoporosis Society	-	<10 ng/mL	10–20 ng/mL	>20 ng/mL		[1] 1–6 months: 3,000 IU orally daily for 8–12 weeks; [2] 6 months to 12 years: 6,000 IU orally daily for 8–12 weeks; [3] 12–18 years: 10,000 IU orally daily for 8–12 weeks; a single or divided oral dose totalling 300,000 units.
14	Pathogenesis and diagnostic criteria for rickets and osteomalacia—proposal by an expert panel supported by the Ministry of Health, Labour and Welfare, Japan, the Japanese Society for Bone and Mineral Research, and the Japan Endocrine Society (74)	2015	Japanese Society for Bone and Mineral Research, Japan Endocrine Society	-	<20 ng/mL	-	-	-	No mention
15	Optimizing bone health in children and adolescents (75)	2014	American Academy of Pediatrics	-	<20 ng/mL	-	≥20 ng/mL	>80 ng/mL	[1] 0–18 years: minimum of 400 IU /day, maximum of 800 IU /day; [2] 2–18 years:50,000 IU quarterly or 80,000–100,000 IU twice in fall and winter;
16	Vitamin D in the Healthy European Paediatric Population (76)	2013	European Society for Paediatric Gastroenterology Hepatology and Nutrition	<10 ng/mL	<20 ng/mL	-	≥20 ng/mL	>96 ng/mL	[1] Infants: 400 IU/day, <1,000 IU/day; [2] 1–10 years: <2000 IU/day; [3] 11–17 years: <4,000 IU/day
17	Practical guidelines for the supplementation of vitamin D and the treatment of deficits in Central Europe - recommended vitamin D intakes in the general population and groups at risk of vitamin D deficiency (77)	2013	Central Europe	-	<20 ng/mL	20–29 ng/mL	≥30 ng/mL	-	[1] 0-6 months:400 IU/day; [2] 6–12 months: 400–600 IU/day; [3] 2–18 years: 600–1,000 IU/day; [4] > 18 years: 800–2000 IU/day; [5] Pregnancy and lactation: 1500–2000 IU/day.
18	Recommended Vitamin D Intake and Management of Low Vitamin D Status in Adolescents: A Position Statement of the Society for Adolescent Health and Medicine (78)	2013	Society for Adolescent Health and Medicine	-	<20 ng/mL	20–29 ng/mL	≥30 ng/mL	>200 ng/mL	[1] Healthy adolescents: 600 IU /day [2] Deficiency or insufficiency adolescents: at least 1,000 IU /day
19	Vitamin D and health in pregnancy, infants, children and adolescents in Australia and New Zealand: a position statement (79)	2013	Australia/New Zealand	<5 ng/mL	5–11 ng/mL	12–19 ng/mL	≥20 ng/mL	>200 ng/mL	[1] Preterm (1) Mild deficiency:Treatment: 200–400 IU/day; Maintenance and prevention:200–400 IU/day; (2) Moderate or severe deficiency: Treatment:800 IU/day; Maintenance and prevention:200–400 IU/day; [2] < 3 months old (1) Mild deficiency:Treatment: 400 IU/day for 3 months; Maintenance and prevention:400 IU/day; (2) Moderate or severe deficiency: Treatment:1000 IU/day for 3 months; Maintenance and prevention:400 IU/day; [3] 3–12 months old (1) Mild deficiency:Treatment: 400 IU/day for 3 months; Maintenance and prevention:400 IU/day; (2) Moderate or severe deficiency: Treatment:1000 IU/day for 3 months, or 50,000 IU stat and review after 1 month (consider repeating dose); Maintenance and prevention:400 IU/day; [4] 1–18 years old (1) Mild deficiency:Treatment: 1000–2000 IU/day for 3 months, or 150,000 IU stat; Maintenance and prevention:400 IU/day or 150,000 IU at start of Autumn; (2) Moderate or severe deficiency: Treatment:1000–2000 IU/day for 6 months, or 3,000–4,000 IU/day for 3 months, or 150,000 IU stat and repeat 6 weeks later; Maintenance and prevention:400 IU/day or 150,000 IU at start of Autumn;

In our study we found that researchers validated study preconceptions in multiple studies on the same research topic. Two studies (30, 31) come from the same research team, experimental studies conducted in 2011 and 2014, respectively. A pilot randomized controlled trial (30) was conducted in 2011 to determine the feasibility, initial safety and efficacy of omega-3 for the treatment of ADHD in children with ASD. After 12 weeks of treatment, there was a correlation between reduced levels of fatty acids and reduced levels of ADHD, and the treatment was well accepted. However there was no statistically significant effect of omega-3 on core symptoms of ADHD or autism. A new, internet-based clinical trial (31) design was conducted in 2014, and the study suggests that internet-based randomized controlled trials of treatments for children with ASD are feasible and may lead to significant reductions in the time and cost of completing a trial. However, omega-3 fatty acids did not lead to a significant reduction in ADHD, but a trend toward a non-significant beneficial effect was observed.

Two studies (20, 39) come from the same research team, experimental studies were conducted in 2016 and 2015.A cross-sectional study was first conducted in 2015 to assess the vitamin D status of individuals with ASD and the relationship between vitamin D deficiency and autism severity. An open trial of vitamin D supplementation in children with ASD was also conducted. The results of the study indicated that vitamin D may be beneficial for individuals with ASD, there was a significant negative correlation between serum 25(OH)D levels and the severity of autism as assessed by CARS scores. Subsequently in 2016, based on the results of the 2015 trial, the effect of vitamin D supplementation on core symptoms of autism in children was further assessed. Using a double-blind, random clinical trial methodology and a more carefully designed study extending from 3 to 4 months in 2015, the results of the study suggest that oral vitamin D supplementation can safely improve signs and symptoms of ASD and can be recommended for children with ASD.

Three studies (43–45) come from the same research team. Two studies (43, 44) were experiments conducted in the same year. A randomized, double-blind, placebo-controlled (43) design to test whether vitamin D and omega-3 were effective in reducing irritability and hyperactivity symptoms in children with ASD. The effect of changes in biomarkers of vitamin D (serum 25(OH)D) or − 3 LCPUFA (omega-3 index) on treatment response was also investigated. Studies have shown that vitamin D and omega-3 can treat irritability symptoms in children with autism, and that vitamin D has a significant benefit on ADHD in these children. The study (44) focusing on, Vitamin D and omega-3 for the treatment of core symptoms of autism in children, the results of the study suggest that supplementation with omega-3 alone or in combination with vitamin D may be effective in treating core symptoms of ASD in children. The study (45) focusing on the alleviation of inflammatory factors in ASD following vitamin D and omega-3 supplementation, findings suggest that vitamin D and omega-3 have the potential to enhance social and communicative functioning in children with ASD, especially when based on known pre-treatment inflammatory conditions.

However, based on this, we are unable to conclude that vitamin D and/or omega-3 supplementation is effective in alleviating ASD, and the results of the omega-3 supplementation studies are individually significant, while the results of the other studies are not significant. Vitamin D supplementation was shown to be effective in improving symptoms, but we were unable to draw firm conclusions based on several experiments, and we are inclined to conclude that vitamin D supplementation improved some of the core symptoms of ASD in patients with ASD, mainly behavioral functioning. However, regarding the experiments on combined vitamin D and omega-3 supplementation, there was a positive effect on ASD, but it was not possible to further identify the starting components. We recommend vitamin D supplementation to improve behavioral functioning in clinical applications.

We need to take into account in our clinical practice the impact of risks such as ethnicity, diet, associated diseases (Hepatic failure, cholestasis, Chronic kidney disease), sun exposure, latitude and longitude of residence on vitamin D levels. Currently, many vitamin D guidelines do not require vitamin D monitoring as part of routine screening, but for many diseases with a high risk of vitamin D, testing is necessary during the disease assessment phase. However, for patients with ASD, many studies have shown that there is a correlation between vitamin D and the development of ASD, and that taking vitamin D can alleviate some of the symptoms. We suggest that when the correlation between vitamin D and the development of ASD is further established (which is where our future research will be directed), vitamin D testing can be added to the ASD screening programme. And it is worth mentioning that in A Central and Eastern European Expert Consensus Statement mentioned that assessing the success of vitamin D treatment after at least 6 to 12 weeks，which is worth guiding us in designing the duration of monitoring in our future studies.

Currently, the main circulating form of vitamin D is 25(OH)D, which has a half-life of 2–3 weeks, and it is the best marker for monitoring vitamin D status. For this reason, blood control for monitoring vitamin D levels is a more idealized means of monitoring, but in practice, frequent blood sampling for patients with ASD is not relatively easy to achieve, and when it is done it is expensive, whether the monitoring is borne by the patient’s family or the hospital. What is more important for us to explore before further blood control is the metabolic pathways, the pathways of action of vitamin D in patients with ASD, and to provide better evidence for the timing of monitoring.

However, this study has its limitations, firstly the small volume of literature included in this study, the small number of subjects relative to the observation group, the fact that the studies were not methodologically consistent with each other, and the risk of assessment bias, despite the fact that we selected multiple individuals with evidence-based experience to judge each other independently when searching the evaluation literature. There is a lack of good quality research on omega-3 in patients with ASD, and there is a lack of standard studies on the timing and dosage of omega-3 supplementation. Regarding the standard omega-3 supplementation dose for patients with ASD, the recommended reference amount, based on evidence-based rationale, is EPA + DHA at a combination of 1.3–1.5 g/day for 16–24 weeks to treat ASD (80). It is recommended that future multicentre studies with large sample sizes be conducted to validate the dose and method of supplementation to promote symptom relief in patients with ASD.

## Conclusion

This review systematically examined the current literature to increase the relief of core symptoms of ASD by omega-3 and vitamin D supplementation. The review found that omega-3 supplementation was weakly effective in improving ASD and was not sufficient to conclude that core symptoms were alleviated. Vitamin D supplementation improved some of the core symptoms of ASD in patients with ASD, mainly behavioral functioning. However, the results of the literature included in this study were slightly mixed. Therefore, we cannot directly conclude that vitamin D supplementation has a beneficial effect on a specific symptom of ASD, but the overall conclusion is that vitamin D supplementation has a positive effect on behavioral functioning in ASD. Omega-3 and vitamin D combination supplementation had good combined effects in patients with ASD, with significant improvements in social and behavioral outcomes. More in-depth studies are needed to explore the mechanisms of action of omega-3 and vitamin D in patients with ASD.

## Data availability statement

The original contributions presented in the study are included in the article/supplementary material, further inquiries can be directed to the corresponding authors.

## Author contributions

YJ and WD: conceptualization and investigation. YJ, WD, and XK: methodology. YJ, WD, and HN: writing—original draft preparation. XK and JG: writing—review and editing. ZJ: supervision. HN: project administration. All authors have read and agreed to the published version of the manuscript.

## Funding

This research was funded by Doctoral Fund of Jiamusi University—Construction and application of gastrointestinal risk prediction model for autism spectrum disorder (JMSUBZ2020-07); Heilongjiang Young Scientist Project (2022QNTJ-016).

## Conflict of interest

The authors declare that the research was conducted in the absence of any commercial or financial relationships that could be construed as a potential conflict of interest.

## Publisher’s note

All claims expressed in this article are solely those of the authors and do not necessarily represent those of their affiliated organizations, or those of the publisher, the editors and the reviewers. Any product that may be evaluated in this article, or claim that may be made by its manufacturer, is not guaranteed or endorsed by the publisher.
